# Towards an Ethical Analysis of Research in One Health (EAROH)

**DOI:** 10.1007/s11673-024-10406-3

**Published:** 2024-12-13

**Authors:** Zohar Lederman

**Affiliations:** Department of Emergency Medicine , Hong Kong University, Pok Fu Lam, Hong Kong

**Keywords:** One health, Animal ethics, Research

## Abstract

The COVID-19 and Monkeypox pandemics and the ongoing Marburg outbreak in Rwanda provide a stark reminder of the importance of espousing a One Health (OH) approach to zoonoses as well as other public health and global health issues. Recent years have in fact seen an exponential rise in biomedical and public health journals and publications explicitly adopting the name of OH. Not all research that pertains to be OH however is indeed OH research, insofar as it does not comply with the proclaimed OH goals of benefiting humans, animals, and the environment. Thus, to ensure such compliance a checklist or toolkit for an ethical analysis of research in OH (EAROH) should be required prior to publication in scientific journals or grant applications. Such a toolkit should be developed by a working group of scholars with expertise in OH ethics, animal ethics, and environmental ethics.

## Introduction

As the COVID-19 and Monkeypox pandemics and ongoing Marburg virus outbreak in Rwanda remind us once again of the threat of zoonoses to global human health, One Health (OH) is prominent on the public health, veterinary, scientific, and political agenda. This is seen particularly in its inclusion in the international instrument on pandemic prevention, preparedness, and response (Pandemic Treaty) and in the fact that it poses one of the main obstacles for wide adoption of the Treaty.^1^ Recent years in general and peri-COVID-19 years specifically have seen an exponential rise in OH publications describing research that proclaims to be OH. Various academic programmes worldwide are preparing the future generations of OH practitioners and researchers, and several peer-reviewed scientific journals now emphasize OH research and/or explicitly carry the title of OH. Considering the proclaimed aim of OH to benefit humans, animals, and the environment (American Veterinary Medical Association 2008), however, not all research that proclaims to be OH is indeed OH research (Lederman 2013; Lederman and Capps 2020).

In what follows, “true” OH research means research that explicitly proclaims to be OH research (either in the title and/or main text of publications) and indeed follows what would be considered as the ideology of OH. While there is certainly no consensus as to the exact content of this ideology, from an ethical perspective it should at the very least include the aim to consider the interests of, and/or benefit non-human entities such as animals and eco-systems. OH research that only proclaims to be OH research but does not really consider the interests of non-human entities is not “true” OH research. This, of course, does not mean that it is “bad” research; it merely means it is not really OH research even if it proclaims to be.

This paper argues for increased scrutiny of publications or grant applications purported to report or support research as OH research. The strategy is as follows. Based on existing publications, this paper initially argues that OH has a strong ethical component—the commitment to benefit humans, animals, and the environment. A further claim is that biomedical and public health research or practices that do not at least aim at any point to benefit humans, animals, and the environment should not be defined as OH. The paper next critically reviews several journals and publications describing research that proclaims to be OH research. Not at all comprehensive, the aim here is merely to glean what researchers themselves consider OH to be, and to assess whether their research may indeed be defined as OH in light of the normative commitment of OH. The last section lays out the first steps towards ensuring that journals, biomedical and public health research, and even research grants that carry the name of OH do not carry it in vain from an ethical perspective. The paper specifically calls to develop a checklist or toolkit for an ethical analysis of research in OH (EAROH) that researchers would have to follow in grant applications and submission for peer-review prior to publication.

The paper does not do two main things. First, it does not mention other kinds of benefits that may be accrued by espousing a OH position. Potential benefits might be financial, social, cultural, political, scientific etc. These have been addressed elsewhere and this paper simply assumes as a truism that espousing a OH approach to public health and global health (and perhaps even in clinical care) may provide benefits that go beyond ethics (Zinsstag et al. 2015, 2006, 2012). Second, the paper does not at all discuss how specific OH research projects or grant applications should be evaluated from an ethical perspective and what sort of ethical guidelines should be drafted and/or applied. These will require the collaboration of multiple stakeholders from industry, political representatives, local communities, civil society in general, and academia. Contributions from academia should be interdisciplinary/ transdisciplinary in nature (Zinsstag 2015; Degeling et al. 2015; Capps et al. 2015).

## The Ethics of One Health

Several bioethicists have discussed the ethical aspects involved in OH for a decade now. They generally perceive OH to have significant ethical implications and commitments. The most basic normative commitment is that OH research, practices, and policies should not focus solely on the interests of human beings. Prioritizing humans while taking into account the interests of non-human beings is one thing, but considering solely the interests of humans when devising and implementing research projects, policies, and practices is first ethically unjustified, and second, really encapsulates the field of public health—so why is there a need for OH? One response usually given is that OH emphasizes interdisciplinary or transdisciplinary collaboration. This, however, reflects a lack of awareness of the public health literature or insight much more than a novel way of thinking that requires a novel approach to global health challenges (Capps et al. 2015; Capps and Lederman 2015a, 2015b, 2016; Lysaght et al. 2017; Lederman and Capps 2018, 2020; Johnson and Degeling 2020; Capps 2022a, 2022b, 2024; Degeling, Lederman, and Rock 2016; Lederman 2016, 2017, 2021; Lysaght et al. 2016; Degeling et al. 2015; Degeling, Dawson, and Gilbert 2019b; van Herten et al. 2019).

Taking seriously this basic normative commitment immediately raises two kinds of foundational questions, one metaethical and one ethical: First, which non-human entities should be considered and why? And second, how may the weighing of human and non-human interests be conducted or evaluated? Answering the first question should at least start with engaging the vast existing literature in environmental philosophy (Clark et al. 2021; Routley 1973; DesJardins 2006). Unfortunately, biomedical scientists writing about OH have generally ignored this literature and this indeed remains of the main gaps in the field of OH. Recent developments at the international level, however, namely the attempt of establishing a global Pandemic Treaty and the creation of a OH High Level Expert Panel may and should motivate bridging this gap. While other attempts at elaborating or at least sketching an ethical framework for OH have existed for some time, this attempt by a group of leading biomedical scientists on behalf of major international organizations is potentially the most authoritative to date and is likely to become known to other biomedical scientists in the field.

Four international Partners, the Food and Agriculture Organization (FAO), the World Animal Health Organization (formally OIE, currently WOHA), the United Nations Environment Programme (UNEP), and the World Health Organization (WHO) convened a One Health High Level Expert Panel (OHHLEP) in 2021. The OHHLEP originally consisted of 26 members from 24 countries. While not having any ethicist on the panel at the time their 2021 summary report makes a remarkable normative assertion as to what OH is all about.

(Quote 1) One Health is an integrated, unifying approach that aims to sustainably balance and optimize the health of people, animals, and ecosystems. It recognizes the health of humans, domestic and wild animals, plants, and the wider environment (including ecosystems) are closely linked and interdependent. The approach mobilizes multiple sectors, disciplines and communities at varying levels of society to work together to foster well-being and tackle threats to health and ecosystems, while addressing the collective need for clean water, energy and air, safe and nutritious food, taking action on climate change, and contributing to sustainable development. (United Nations High-Level Expert Panel 2021, 13)

The OHHELP first establishes OH as an approach that aims to balance and optimize the health of humans, animals, and ecosystems. It recognizes the interconnectivity between environment, animals, and humans, particularly when it comes to health, meaning that ill-health in animals may affect humans and vice versa, and that what is loosely (albeit inaccurately) referred to ill-health of the environment (Selter and Salloch 2023) may also affect humans and animals and vice versa. Interconnectivity also means that animals and humans often share similar needs, and supplying such needs often require healthy environments. The OHHLEP also calls for interdisciplinarity.

More profoundly, the OHHLEP establishes the following assertion as a foundational principle of OH:

(quote 2) Socio-ecological equilibrium that seeks a harmonious balance between human—animal-environment interaction and acknowledging the importance of biodiversity, access to sufficient natural space and resources, and the intrinsic value of all living things within the ecosystem. (United Nations High-Level Expert Panel 2021, 14)

All living things have an intrinsic value. By “intrinsic value” the panel means moral value that does not depend on the instrumental value of all living things, meaning that does not depend on whether or not these living things contribute to some other goal that is valuable in and of itself (Kegan 1998). If you possess intrinsic value, then you matter morally regardless of what you mean to other creatures. Individual flies, then, have moral value regardless of the role they play in ecosystems—even if ecosystems did not depend on flies or benefit from them in any way, flies would still possess moral value.

Another foundational principle asserts human responsibility to ensure animal welfare as well as the integrity of the whole ecosystem, (quote 3) “…thus securing the wellbeing of current and future generations” (United Nations High-Level Expert Panel 2021, 14).

Quote 3 may be interpreted as anthropocentric, as in saying that humans should care for the welfare of animals and the integrity of whole ecosystems only insofar as it benefits humans. Quote 1 may also be interpreted in a similar vein, as in saying that humans should care about the health of animals, plants, and the wider environment only insofar as humans care about their own health, considering that human health and animal and environmental health are interconnected. There is no mistaking about quote 2, however, and its biocentric orientation. The OHHLEP has not elaborated on this bold assertion that all living creatures possess moral value although a peer-review publication is promised and it may clarify the OHHLEP’s ethical position reflected in quote 2. Reflecting on the two foundational questions mentioned above, asserting that life qua life matters morally is a meta-ethical claim. As such, it only tells us whose interests should be considered but says nothing about the way in which these interests should be weighted. And even as a meta-ethical claim, it requires an explanation, an argument—why is the fact of merely being alive grants one moral value?

For the time being, several assumptions may be deduced. First, the ethical position reflected in quote 2 not only reflects the position of the OHHLEP but by proxy all the four partners that sponsor the OHHLEP: the WHO, UN, WOHA, and the FAO. Moreover, quotes 1,3 may now be interpreted in light of quote 2: What matters is the well-being of animals and sustainability of ecosystems in addition to that of humans; clean water, energy and air, and safe and nutritious food are needed for the collective that consist of humans, animals, and the environment. What matters is not only current generations but also future generations of humans, animals, and ecosystems.

If this is indeed the position of the OHHLEP (and the four partners), then it is both strong and bold. It reflects a strong normative position, meaning a position of how we ought to behave. It is strong because it is demanding: the OHHLEP seems to suggest that since all living creatures have intrinsic value they should be respected prima facie. To respect something means first of all to avoid harm or to strive to avoid harm, and prima facie means that you should not harm something unless other justifications compel you to do so. The OHHLEP is in fact asserting that humans should not harm animals or ecosystems unless they must do so for other reasons. A recent *Lancet* editorial strengthens the positions adopted by the OHHLEP: “The consequences of this thinking entail a subtle but quite revolutionary shift of perspective: all life is equal, and of equal concern” (The Lancet 2023, 169), going the extra length of even recommending plant-based diet over animal-based diet.

Such a strong moral commitment may be advanced casually by scientists who do not feel the need to elaborate and defend it—and it happened before (Goldberg and Patz 2015)—but for (bio)ethicists it is just a beginning of a conversation; a very lengthy and tedious conversation.

Biocentrism, or the ethical position that all living creatures possess intrinsic moral worth, is a well-known but highly controversial theory in environmental ethics (Agar 2001; Basl 2019). In modern times, Albert Schweitzer is the philosopher most associated with biocentrism. Schweitzer argued that life qua life matters morally, as all living creatures possess a “will-to-live” or a determination to live (Schweitzer 1987). Schweitzer argues that

For our conception of the universe and of life, then, the gain derived from knowledge is only that it makes it harder for us to be thoughtless, because it ever more forcibly compels our attention to the mystery of the will-to-live which we see stirring everywhere. (Schweitzer 1987, 308)

By this Schweitzer means that humans, acknowledging their own will-to-live, cannot but acknowledge the will-to-live of other creatures. And insofar as the will-to-live in humans grants them intrinsic moral value, then the same will-to-live in other living creatures grant them intrinsic moral value as well. Once we as rational, thinking, moral agents or persons realize this, we must act accordingly.

Again, for ethicists, this position raises eternal questions and dilemmas. First, Schweitzer, following Asian traditions, considered plants and even grass to be alive. The OHHLEP explicitly refers to plants but it is unclear whether plants bear considerations because they are part of the ecosystem or as living creatures. Whether plants are indeed alive is a philosophical question which depends in part on scientific data. Science so far tells us that plants have a nervous system which allows them to communicate with one another and with other creatures, and to experience and respond to the external environment using a sensory system that may be called sight and smell (Calvo et al. 2017; Baluška and Mancuso 2020; Wohlleben 2016). Whether plants are alive however, or even sentient, is still an area of great controversy.

More urgently, even if we agree that all living creatures have intrinsic moral value, and all possess a will-to-live, meaning that all living creatures have an interest in surviving and prospering, we still need to decide whose interests matter more. Oftentimes in public health and public policy value judgments must be made regarding whose interests should be prioritized over the interests of others in specific contexts. One stark example is the culling of farm, pet, or wild animals in response to an infectious disease outbreak like the culling of badgers in response to bovine Tuberculosis in England (Lederman 2016) or mink in response to COVID-19 in Europe (Lederman 2022). As in culling, each case will present different considerations and challenges, and professional ethicists as well as other stakeholders including civic society would have to be consulted.

Even ignoring the latter difficulty, the position that all living creatures will be hard to digest because it may have extreme or counter-intuitive ramifications. Science tells us that bacteria are living creatures—do they possess intrinsic moral value as well (Cockell 2011)?

Whoever claims that all living creatures have intrinsic value, then, has their work cut out for them. Regardless of the rough edges, however, the OHHLEP and the four partners are explicitly committed to the claim that life qua life matters morally and that it is a foundational principle of OH. At the very least, this means that OH research, practices, and policies should be based on that normative position. In other words, researchers and policymakers who wish to link their research or public policy to OH may do so only insofar as their research and policy remain loyal to the normative commitment that all living creatures have intrinsic moral value. Otherwise, they cannot claim that their research or policy represents OH or is done in the spirit of OH. Alternatively, they must admit that they do not accept the definition of OH advanced by the OHHLEP and the four partners. In this case, they might be expected to explain why. Further, they might be expected to articulate another argument for the moral value of non-human entities. In cany case, the main point is that if they proclaim to be doing OH they should clearly explain or understand themselves why they wish to benefit animals and eco-systems—is it strictly to benefit humans eventually or also some non-human animals?

The next section asks yet another question: do OH journals and OH publications currently follow such a biocentric normative commitment? Or in other words, does OH research currently remain faithful to what the OHHLEP identified as foundational core normative values?

## Searching for Ethics in One Health Publications

Several biomedical and public health journals now explicitly mention OH either in their title or their agenda: *One Health*,^2^*The International Journal of One Health*,^3^*Ecohealth*,^4^*Science in One Health*,^5^*One Health & Implementation Research*,^6^*Ecosystem Health and Sustainability*,^7^ and others. Their proclaimed mission obviously reflects how the journal editors perceive OH, and it may influence authors in their thinking and writing about OH. The journal *One Health* seems to be merely interested in “inter- and intra-species pathogen transmission,” and the *International Journal of One Health* does not seem to have a mission at all. The journal *Science in One Health* aims to “reduce the inequity of health issues in the world and promote more advanced researches (sic) on One Health.” Inasmuch the two are comparable, there is undoubtedly tremendous health inequity between humans and animals in the world, for instance in specific countries’ and global allocation of resources devoted to human and animal health, and the ability of animals and humans to reach the full potential of their life span. The journal, however, more likely refers to health inequity among humans. Inequity among humans undeniably merits attention, but is not specific to OH. In other words, addressing health inequities in humans does not particularly require the insights and methodology found in and encouraged by OH. The *Journal of One Health & Implementation Research* aims “to promote multidisciplinary and transdisciplinary research that provides evidence to improve health for all.” By “all” the journal seems to mean “people, wildlife, and domestic animals,” but it is left unstated why the health of animals is important—whether it is for their own sake or to reduce disease in humans. Lastly, the journal *EcoHealth* proclaims to be “a central platform for fulfilling the mission of the *EcoHealth* Alliance to strive for sustainable health of people, domestic animals, wildlife, and ecosystems by promoting discovery, understanding, and transdisciplinarity.” Again, it is not stated why the sustainable health of animals and ecosystems is important.

OH journals then do not seem aware or concerned about the normative commitment entailed by OH as argued above. Can such a commitment be found in OH publications? A definite response to this question will require a systematic review of OH publications, which was not conducted for the purposes of this paper. Having some familiarity with the literature, however, leaves little room for doubt that the normative commitment is hardly acknowledged or used to guide research. Merely for illustration, three OH publications are briefly reviewed below.

In one study Israeli researchers implanted human intestinal grafts in the backs of eight-three immunosuppressed mice in order to test the potential etiologic role of Mycobacterium Avium Paratuberculosis (MAP) in Crohn’s disease. They euthanized the mice at 13–16 weeks post implantation (Golan et al. 2009). This study is considered by Israeli authors as an Israeli experience of OH (Klement et al. 2009).The same authors however claim that OH strives to gain “mutual benefit of all,” seemingly referring to humans, animals, and the environment. Unfortunately, the study did not benefit those eight-three mice or mean to benefit any other animal in general. The study described seems to be a rather standard biomedical study involving the use of mice, without any relevance to OH or vice versa.

A more recent example may be gleaned from a study that reviews the OH infrastructure in Jordan. The authors of this study highlight the interdisciplinary nature of OH but lament the lack of involvement of the Ministry of Environment. The focus of the study seems to be strictly human health (Abutarbush et al. 2022).

Yet another example may be seen in a recent editorial by two prominent OH scientists for a special issue on OH (Mackenzie and Jeggo 2019)—while interdisciplinarity is emphasized, there is no mention of the normative commitment of OH.

A similar gap is experienced in most OH scientific gatherings, at least in the experience of the present author.

The normative commitment of OH then is mostly absent from current OH publications and agenda (see also Lederman 2021). This may not be an issue as long as we knowingly and explicitly ignore the normative commitment of OH and the OHHLEP’s mission. But if the latter is to be reckoned with, then something must change in OH publications. How can researchers then ensure and promote biomedical and public health research that is indeed OH research?

## Emphasizing Ethics in One Health Publications and Grants

Davis et al. have developed the Checklist for One Health Epidemiological Reporting of Evidence (COHERE), suggesting nineteen standards to guide OH research design, analysis, and reporting. According to them, OH research should address or involve all three “stakeholders.”

Ethics is mentioned three times: 1) in regards to human subjects research: “Ensure human subject assurances adhere to the highest standards of ethics governing human subjects research.” 2) in regards to animals subject research: “Ensure animal subject assurances adhere to the highest standards of ethics governing animal subjects research” and 3) as a standalone standard which details the following two items: 3a) “Report animal (IACUC/ACUC) and human ethics (IRB) approvals, as well as other relevant permissions that were obtained”; 3b) If applicable, describe the framework for adhering to community based research standards (e.g. community approval, cultural respect, knowledge translation) (Davis et al. 2017).

This paper seeks to raise an awareness among the scientific community of the ethics involved with the concept of OH. As been already demonstrated in the bioethics literature (Capps and Lederman 2015b, 2016; Capps 2022a; van Herten et al. 2020; Buse et al. 2018), OH ethics involves much more than what is expected by Davis et al. OH ethics certainly includes aspects of human and animal research ethics, as well as public health and global health ethics, but it goes far beyond them, examining culling practices for instance or the use of sentinel animals (Lederman 2021). OH establishes a scientific and ethical imperative to collaborate in order to benefit humans, animals, and the environment. Implementing the scientific imperative requires robust statistical analysis, which often requires a biostatistician accompanying the research project from the design stage to publication. Taking seriously the ethical imperative of OH would similarly require the inclusion of bioethicists throughout the process of research and OH policymaking and implementation. Biomedical and public health journals would then demand an ethical analysis of OH research as a prerequisite for consideration. Funding organizations as well would withhold support for OH research unless a preliminary ethical analysis accompanies the grant application, assessing the extent to which the study fulfils the normative commitment of OH as sketched above.

In order to streamline and standardize such a process in the work of OH researchers and policymakers, particularly if bioethicists are not formally involved, a toolkit used by authors may be beneficial.

## Developing an Ethical Analysis Toolkit to Assess OH Research

The Nuffield Council on Bioethics, in its report on the ethics of research involving animals, recommends “ … that all journals publishing results of research involving animals consider the inclusion of a category on the Three Rs in the methodology section” (Nuffield Council on Bioethics, 275). The Council also recommends that “ … funding bodies should request that for each project that receives funding, a short summary be submitted… which describes the way in which the Three Rs were implemented in the project …” (Nuffield Council on Bioethics, 275) (the three Rs mean Reduction, Refinement, and Replacement).

The proposal here is similar in spirit. Currently, research involving humans and/ animals must include an approval by an institutional ethics committee, specifically designated to protect the welfare and rights of humans or animals respectively. These committees, however, do not assess whether research that pertains to be OH indeed adheres to OH normative principles as stated above. The following suggestion, then aims to complement or be an add on to the ethics approval process already in place.

OH and general biomedical or public health journal editors should require an elaborate ethics section as a necessary condition for publication, specifically meant to assess compliance with OH ethics. This may mean that a third reviewer, with expertise in animal ethics, environmental ethics, or OH ethics, would be invited to review the manuscript in addition to two reviewers with relevant scientific expertise. Additionally, a more robust ethics analysis should be added to the COHERE ethics standard. Specifically, criterion 3b described above should require description of the ethical framework: how do the authors perceive the moral status of all the potential or actual stakeholders in their study, including animals and the environment? Based on this, the authors should then justify the harm or potential harm effected on these stakeholders.

At the very least, authors should be explicit about the specific normative grounding of their study or research proposal: is it anthropocentric, ecocentric, or biocentric (Cafaro and Primack 2013)? If their submission does not follow the normative commitment of OH as expressed by the OHHLEP, researchers or grant applicants should clearly explain their reasoning. A useful application in this regard may be a OH ethics checklist or toolkit which includes a set of standard, well-devised queries. Examples include the following:

1. Does the study entail potential risks for the local ecosystem?
2. Does the study bear any potential benefits for non-human animals?
3. Are there any potential interventions in addition to the one investigated that may be non-inferior in effect but likely to be less harmful towards the local ecosystem or non-human animals?

Researchers will have to respond to these queries during the process of submission or as a condition for grant application. The result will be some sort of a pictorial representation, such as a Venn diagram, that would roughly quantify to what extent the study is anthropocentric, ecocentric, or biocentric. Based on this assessment, journal editors, reviewers, and funders could decide whether the study is indeed a OH study, and whether it passes ethical scrutiny.

The process of developing such an Ethical Analysis of Research in OH (EAROH) would require some effort. It would be reasonable to gather an international working group of senior and junior bioethicists and philosophers, with expertise in environmental ethics, animal ethics, climate change ethics, and particularly OH ethics. This group will be tasked with developing a set of queries that would be presented to researchers. An algorithm would then be developed to synthesize the answers to these queries and present them in a quantitative, pictorial manner, e.g. Venn diagram. If risks and benefits were not balanced equally or roughly equally among the stakeholders, researchers would then have to justify the prioritization of one over the others. The quantitative summary, along with the researchers’ justifications, would then be submitted to the journal or funding body in addition to the scientific content.

In light of considerate empirical work already done in the context of OH and public attitudes toward potential OH interventions (Degeling, Thomas, and Rychetnik 2019a, b; Degeling et al. 2017a, b; Johnson et al. 2019), the working group is likely to benefit from surveys of both researchers and publics prior to the development of said queries. Such surveys would explore how researchers and community members interpret the ethical obligations associated with their work, their understanding of concepts such as ecocentrism and biocentrism and their attitudes towards OH in general.

Importantly, EAROH should not be used to judge whether a specific research project is ethically justified—researchers are likely to respond negatively to such critique from perceived outsiders, and this may create the opposite effect. Rather, EAROH would aid researchers to ensure that their research indeed corresponds with the proclaimed aims of OH and the normative commitment as advanced by the OHHLEP, thus ensuring optimal benefits for humans, animals, and the environment.

What are the potential benefits of developing and implementing EAROH? First, it would encourage researchers to think more deeply about the ethical aspects of their research, particularly as it pertains to ethics that go beyond the human. Second, assuming that OH indeed represents an ethical perspective or orientation that is different from traditional public health or biomedical science, EAROH will allow researchers to reflect on whether their research or grant application truly belong to the field of OH. Again, this would say nothing about the quality of their research—it would simply confirm whether their research is what it proclaims to be. Third, and relatedly, using EAROH will facilitate a better understanding and lead to a clearer definition and vision of OH. Currently, as partially demonstrated above, the rapidly expanding OH literature suffers from what might be described an equivocation fallacy—using the same term with substantially different meanings. The term OH, in this sense, is often used to denote nothing more than traditional public health with the same ethical commitments as public health—to promote human health and well-being. There is nothing wrong with this commitment and mission, but OH has commitments that extend beyond the human. Using the term OH where it is appropriate and avoiding using it when it is inappropriate would help clean the field of OH and prevent confusion among students, researchers, policymakers, and other stakeholders.

## Conclusion

Increasing resources worldwide are being channelled to OH research with several biomedical and public health journals now carrying the banner of OH and an exponential rise in OH publications. Several bioethicists have been engaging with the idea and practice of OH, exploring ways to promote biomedical and public health practice and research that is congruent with the ethical implications of OH. The recent OHHLEP report puts the wind in their sail.

The proposal here is a further step towards aligning the science of OH with its ethics. It calls for the development of an ethical analysis of research in One Health (EAROH) toolkit, to be used by researchers, journal editors, reviewers, and funders to assess the ethics and compliance with OH normative commitments of research that proclaims to be OH research. The proposal calls for the establishment of an interdisciplinary, international, working group for this purpose.

## Data Availability

Not applicable.

## Footnotes

1 Intergovernmental Negotiating Body to draft and negotiate a WHO convention, agreement or other international instrument on pandemic prevention, preparedness and response. Report by the Director-General 2024.

2 https://www.sciencedirect.com/journal/one-health

3 https://www.onehealthjournal.org/

4 https://www.springer.com/journal/10393

5 https://www.journals.elsevier.com/science-in-one-health

6 https://ohirjournal.com/

7 https://www.tandfonline.com/toc/tehs20/current
